# Preoperative risk stratification in endometrial cancer using ESGO/ESTRO/ESP 2021 guidelines: accuracy with and without molecular classification

**DOI:** 10.1186/s12885-025-14741-5

**Published:** 2025-08-11

**Authors:** Petra Bretová, Munachiso Iheme Ndukwe, Jan Laco, Hana Vošmiková, Taťána Rešlová, Denisa Pohanková, Klára Balcarová, Jiří Haviger, Jana Marie Havigerová, Igor Sirák

**Affiliations:** 1 Department of Obstetrics and Gynecology, Charles University, Faculty of Medicine in Hradec Kralove, Hradec Kralove, Czech Republic; 2 Department of Oncology and Radiotherapy, University Hospital Hradec Kralove, Hradec Kralove, Czech Republic; 3 The Fingerland Department of Pathology, Charles University, Faculty of Medicine in Hradec, University Hospital Hradec Kralove Kralove, Hradec Kralove, Czech Republic; 4 Department of Non-medical Studies, Faculty of Medicine in Hadec Kralove, Charles University, Hradec Kralove, Czech Republic; 5 Department of Informatics and Quantitative Methods, Faculty of Informatics and Management, University of Hradec Kralove, Hradec Kralove, Czech Republic; 6 Department of Preventive Medicine, Faculty of Medicine in Hradec Kralove, Charles University, Hradec Kralove, Czech Republic

**Keywords:** Endometrial cancer, Molecular classification, Sentinel node biopsy, Risk stratification, Ultrasonography, Next-generation sequencing, ESGO/ESTRO/ESP 2021 guidelines

## Abstract

**Background:**

The study aimed to evaluate the impact of integrating molecular classification with imaging-based preoperative staging on risk stratification prediction in endometrial cancer patients in accordance with ESGO/ESTRO/ESP (European Society of Gynaecological Oncology/European Society for Radiotherapy and Oncology/European Society of Pathology) 2021 guidelines.

**Methods:**

A retrospective cohort of 143 endometrial cancer patients was analyzed to assess changes in preoperative risk stratification after incorporating molecular classification into clinical evaluation. Preoperative clinical staging was primarily based on transvaginal ultrasound imaging. The overall agreement between preoperative risk group estimates (with/without molecular classification) and final postoperative outcomes was assessed using weighted Cohen’s Kappa, with bootstrap 95% confidence intervals and quadratic weights.

**Results:**

The addition of molecular classification significantly improved preoperative risk stratification accuracy (from 59.4 to 73.4%), particularly for patients post-operatively classified as high-risk. Kappa values indicated an improvement in overall agreement between preoperative and postoperative risk stratification following the addition of molecular classification, from 0.551 (95% CI: 0.430–0.671) to 0.767 (95% CI: 0.675–0.849). The non-overlapping confidence intervals indicated statistical significance. Preoperative assessment without molecular input tended to underestimate risk stratification. However, 26.6% of patients remained misclassified due to other factors, mostly within the intermediate and high-intermediate risk groups.

**Conclusions:**

Incorporating molecular classification enhances preoperative risk stratification and has the potential to tailor surgical treatment. Further validation through prospective multicentric studies is needed to support our findings.

**Supplementary Information:**

The online version contains supplementary material available at 10.1186/s12885-025-14741-5.

## Background

Endometrial cancer remains the most common gynecological malignancy in developed countries, with incidence rates showing no signs of decline in recent years [1]. In the past decade, significant progress has occurred in clinical practice, particularly with the adoption of molecular classification (MC) and sentinel lymph node biopsy (SLNB). The 2021 ESGO/ESTRO/ESP (European Society of Gynaecological Oncology/European Society for Radiotherapy and Oncology/European Society of Pathology) guidelines introduced updated risk stratification categories—low, intermediate, high-intermediate, and high-risk of recurrence—enabling more tailored treatment approaches [2].

Preoperative risk stratification guides surgical decision-making and is traditionally based on histological type, tumor grade, and disease stage determined through expert ultrasonography or MRI (magnetic resonance imaging), without molecular classification. Accurate imaging is essential for evaluating myometrial and cervical invasion. In cases of sentinel node biopsy failure, particularly among high-intermediate and high-risk patients, side-specific lymphadenectomy is recommended for accurate nodal staging [2]. Conversely, in low and intermediate-risk patients, particularly those without myometrial invasion, lymph node staging may be safely omitted in case of SLNB failure.

In clinical practice, postoperative risk classification incorporates definitive histological findings, including LVSI (lymphovascular space invasion), alongside molecular features, which may alter risk categories and guide adjuvant therapy. For example, a low-grade, FIGO (International Federation of Gynecology and Obstetrics) 2009 stage IA tumor (initially low-risk) may be upgraded to high-risk if a *TP53* (Tumor protein 53) mutation is found postoperatively, while high-intermediate risk tumors with cervical invasion may be downgraded if a *POLE* (Polymerase ε) mutation is identified [2]. Molecular classification refines the use of chemotherapy, radiotherapy, or hormonal therapy, ensuring tailored treatment. High-risk patients benefit from intensified therapy, while low-risk patients avoid unnecessary interventions. Moreover, molecular classification improves the prediction of treatment response [3].

This study aimed to evaluate whether incorporating MC into the preoperative staging of apparent early-stage (FIGO 2009 stages I-II) endometrial cancer improves risk stratification according to the ESGO/ESTRO/ESP guidelines. The study also assessed whether preoperative risk stratification without MC is more susceptible to underestimation or overestimation.

## Methods

### Patients and clinical data

This retrospective observational single-center study was conducted at the Gynecological Oncology Center of University Hospital Hradec Kralove, Czech Republic. Patients who underwent surgical treatment for histologically confirmed endometrial cancer (EC) between January 2022 and December 2024 were consecutively included.

Inclusion criteria included: apparent early-stage endometrial cancer (FIGO 2009 stages I-II), available histology from biopsy (dilatation and curettage or hysteroscopy) and definitive hysterectomy, preoperative staging by expert ultrasound, pelvic-abdominal-chest CT (computed tomography; waived in low-risk cases), and immunohistochemistry (IHC) for p53, mismatch-repair proteins (MMR), as well as next-generation sequencing (NGS) analysis for *TP53* and *POLE* mutations (waived in low-risk cases).

Exclusion criteria included patients with synchronous malignancies, apparent advanced-stage disease (FIGO 2009 stages III–IV), or missing essential data (molecular classification, imaging results). We also excluded patients without confirmed malignant histology from the preoperative biopsy—these were cases where only atypical hyperplasia/benign histology was reported preoperatively or where no biopsy was performed at all (e.g., surgery was indicated for other reasons such as fibroids or uterine prolapse, and endometrial cancer was diagnosed incidentally in the hysterectomy specimen).

Data collected included: age, BMI (body mass index), ultrasound and CT findings (depth of myometrial invasion, cervical involvement, lymphadenopathy, distant metastases), and histopathological details from biopsy and final histology (FIGO 2009 stage, histological type, grade, LVSI, IHC results for MMR proteins, p53, and NGS results for *POLE* and *TP53*). All data were extracted from the hospital’s information systems.

### Preoperative imaging

All patients underwent transvaginal and transabdominal ultrasound staging conducted by a certified ultrasonography expert (T.R.) evaluating tumor extent, myometrial and cervical involvement, and lymph nodes, using IETA (International Endometrial Tumor Analysis) terminology and Czech national guidelines [4, 5]. Pelvic-abdomen-chest CT was performed for non-low-risk cases.

### Histopathology, immunohistochemistry and next-generation sequencing

The tissue specimens were fixed in formalin and processed routinely for histopathology. All endometrial carcinomas were initially diagnosed and/or finally reviewed and confirmed by an expert gynecologic pathologist (J.L.), following the WHO (World Health Organization) recommendations and international standards [2, 6–9]. All tumors were tested for p53 protein and MMR proteins (MLH1, MSH2, MSH6, and PMS2) expression using IHC.

For NGS, DNA was extracted from formalin-fixed and paraffin-embedded (FFPE) tumoral tissue using the Cobas DNA Sample Preparation Kit (Roche Diagnostics GmbH, Mannheim, Germany). Subsequently, NGS-based technology was performed (Hybrid capture DNA Roche KAPA Evo Plus Kit, Roche Sequencing Solutions, Inc., USA) using a custom panel that targets the exons and flanking sequences (+/- 20 bp) of 96 cancer-related genes, including *TP53*,* POLE* genes, and 49 microsatellite regions for MSI status assessment.

Finally, all tumors were classified as POLEmut (*POLE* mutated), MMRd (mismatch-repair deficiency), p53abn (p53 abnormal), or NSMP (non-specific molecular profile).

Details are described in Additional file 1.

### Preoperative and postoperative risk stratification

Patients were preoperatively classified according to the ESGO/ESTRO/ESP 2021 guidelines [2] based on histological type and grade, as well as clinical stage derived from imaging. The tumor board then recommended surgical management, typically consisting of hysterectomy with bilateral salpingo-oophorectomy and either SLNB or systematic lymphadenectomy. SLNB was preferred for patients with low- and intermediate-risk profiles, while systematic lymphadenectomy was performed in cases preoperatively assessed as high–intermediate- or high-risk. In selected low-risk cases without myometrial invasion, lymph node staging was omitted.

Postoperative risk stratification was reassessed using definitive histology, including the presence of LVSI, IHC, and NGS when indicated. *POLE* testing was not routinely performed in low-risk cases, as it would not influence risk categorization or treatment decisions [9].

To explore the potential improvement in preoperative risk stratification, we developed a modified model by integrating molecular classification with standard preoperative clinical and histopathological features. This allowed us to assess the impact of molecular classification on the accuracy of initial risk assessment.

### Statistical analysis

Three risk stratification conditions were evaluated: (1) preoperative classification based on clinical and histopathological features without molecular classification; (2) preoperative classification including molecular classification; and (3) final postoperative classification based on final histopathological type, grade, pathological staging, LVSI, and molecular classification, all non-normally distributed (Shapiro-Wilk, *p* < 0.05).

Agreement between preoperative risk stratification (with and without MC) and postoperative risk stratification was assessed using Cohen’s Kappa with 95% bootstrap confidence intervals. Kappa values were interpreted according to Fleisss et al. [10]: 0.41–0.60 moderate, 0.61–0.80 good, > 0.80 excellent.

To assess if preoperative risk stratification without MC were more prone to underestimation or overestimation compared to those with MC, misclassification patterns were analyzed by categorizing errors in the confusion matrix as underestimation (risk of recurrence rated below the postoperative risk group) and overestimation (risk of recurrence rated above the postoperative risk group).

Prediction performance across risk levels was assessed using multiclass confusion matrix metrics, including precision (proportion of correct severity estimates among those assigned), recall (proportion of actual severity cases correctly identified), and F1-score (harmonic mean of precision and recall, balancing both). Values > 0.70 are considered acceptable, and > 0.80 strong in medical diagnostics [11].

### Software

Data processing and statistical analyses were performed using Python (version 3.10.15) with the following packages: pandas (2.1.4) for data manipulation, numpy (1.25.2) for numerical operations, statsmodels (0.14.4) for statistical modeling, sklearn (1.1.3) for machine learning metrics, scipy (1.9.3) for scientific computations, plotly (6.0.0) and matplotlib (3.6.3) for interactive and static visualizations, and seaborn (0.13.2) for enhanced statistical graphics.

## Results

**Fig. 1:**
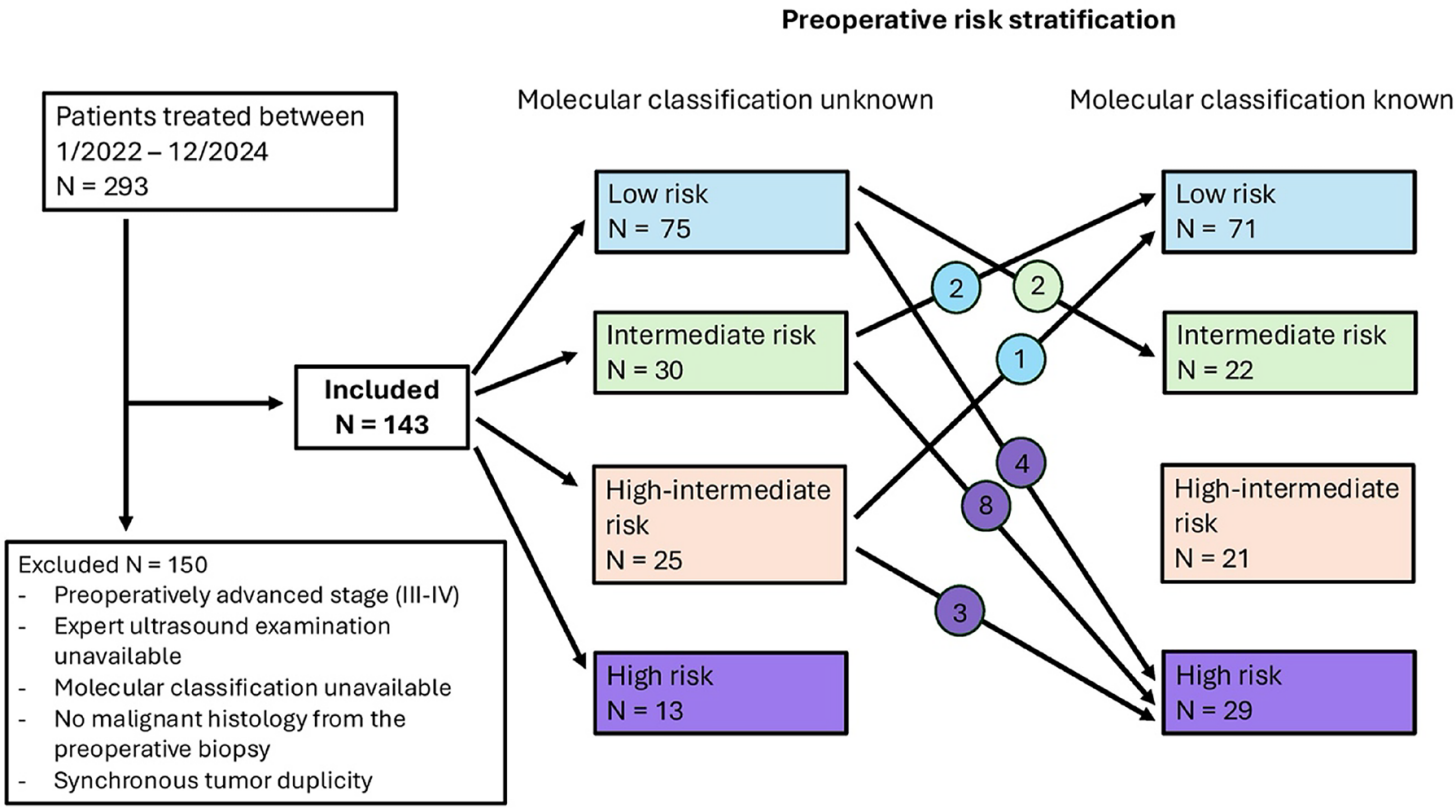
Flowchart of patients included in the study

During the study period, 293 patients were diagnosed with endometrial cancer at our institution. Of these, 143 patients met the inclusion criteria and were included in the final analysis (Fig. 1). The median age was 66 years (mean 64, range 29–85), and the median BMI was 32 kg/m² (mean 33, range 20–56). The majority of patients 97 (67.8%) underwent SLNB, followed by systematic lymphadenectomy 27 (18.9%). In 19 (13.3%) patients, lymph node staging was omitted due to a preoperatively assessed low-risk tumor without myometrial invasion. Most tumors were endometrioid (89.5%) and low grade (81.1%). According to the final staging, 75.5% of patients had FIGO 2009 stage I disease. Detailed histopathological characteristics are displayed in Table 1. Based on molecular profiling, 6 cases (4.2%) were *POLE*mut, 30 (21.0%) MMRd, 27 (18.9%) p53abn, and 80 (55.9%) NSMP. Five tumors exhibited features of multiple molecular classifiers and were assigned according to the recommended hierarchy. Detailed methodology is provided in Additional file 1.

**Table 1:** Baseline clinicopathologic characteristics of the study cohort (*n* = 143)

Variable	Category	*n* (%)
Histologic tumor type	Endometrioid	128 (89.5%)
	Serous	8 (5.6%)
	Carcinosarcoma	4 (2.8%)
	Undifferentiated/dedifferentiated	2 (1.4%)
	Clear cell	1 (0.7%)
Tumor grade	Low grade	116 (81.1%)
	High grade	27 (18.9%)
Cervical stromal invasion	Absent	116 (81.1%)
	Present	27 (18.9%)
Myometrial invasion	None	1 (0.7%)
	< 50%	88 (61.5%)
	≥ 50%	54 (37.8%)
Lymphovascular space invasion	Absent	111 (77.6%)
	Focal	11 (7.7%)
	Substantial	21 (14.7%)
Surgical FIGO 2009 stage	IA	73 (51.0%)
	IB	35 (24.5%)
	II	17 (11.9%)
	IIIA	4 (2.8%)
	IIIB	2 (1.4%)
	IIIC	10 (7.0%)
	IVA	0 (0.0%)
	IVB	2 (1.4%)
Molecular classification	NSMP	80 (55.9%)
	MMRd	30 (21.0%)
	p53abn	27 (18.9%)
	POLEmut	6 (4.2%)

The distribution of ESGO/ESTRO/ESP 2021 risk groups across the three assessment stages is shown in Table 2.

**Table 2:** Distribution of ESGO/ESTRO/ESP 2021 risk groups across assessment stages

Risk Group	Preoperative without MC	Preoperative with MC	Postoperative
Low risk	75 (52.4%)	71 (49.7%)	61 (42.7%)
Intermediate risk	30 (21.0%)	22 (15.4%)	25 (17.5%)
High-intermediate risk	25 (17.5%)	21 (14.7%)	16 (11.2%)
High risk	13 (9.1%)	29 (20.3%)	41 (28.7%)

*MC* Molecular classification

Without molecular classification, only 59.4% of EC patients were preoperatively classified into the correct risk group. The incorporation of molecular classification enhanced the accuracy of preoperative risk assessment in 14.0% of cases, increasing correct stratification from 59.4 to 73.4% of EC patients. However, a total of 26.6% of EC patients remained misclassified due to inaccurate preoperative assessment of cervical invasion (9.0%), myometrial invasion (6.3%), advanced-stage disease (4.2%), LVSI (3.5%), nodal metastases (2.8%), and tumor grade (0.7%).

Cohen’s Kappa coefficient for agreement between preoperative and postoperative risk classification improved from 0.551 (95% CI, 0.430–0.671) without molecular classification to 0.767 (95% CI, 0.675–0.849) with molecular classification, indicating an improvement from moderate to good agreement. As the 95% confidence intervals did not overlap, this difference was statistically significant. Inclusion of molecular classification improved agreement from moderate to good. Confidence intervals did not overlap, indicating statistical significance.

The results of risk group prediction success aggregated into categories of correct, underestimate, and overestimate are shown in Table 3.

**Table 3:** Prediction success rates for preoperative estimates by the categories of correct predictions, overestimation, and underestimation

Category	Without MC (CI)	With MC (CI)
Correct	0.587 (0.505–0.665)	0.706 (0.627–0.775)
Overestimate	0.091 (0.054–0.149)	0.077 (0.043–0.132)
Underestimate	0.322 (0.251–0.402)	0.217 (0.157–0.291)

*MC* Molecular classification, *CI* confidence interval

Notable shifts of more than 0.1 were observed in the correct and underestimation categories, though changes were not statistically significant (confidence intervals overlapped).

A detailed comparison of prediction accuracy for risk stratification (low risk, intermediate risk, high-intermediate risk, and high risk), using the metrics precision, recall, and the overall F1-score, is presented in Table 4. Bootstrap confidence intervals for each value were computed and they are presented in Additional file 2 for better clarity.

**Table 4:** Prediction accuracy for risk stratification (low risk, intermediate risk, high-intermediate risk, and high risk)

Grade	Precision		Recall			F1	
	Without MC	With MC	Without MC		With MC	Without MC	With MC
Low risk	**0.720**	**0.789**	**0.885**		**0.918**	**0.794**	**0.848**
Intermediate risk	0.367	0.500	0.440		0.440	0.400	0.468
High-intermediate risk	0.240	0.286	0.375		0.375	0.293	0.324
High risk	**1.000**	**0.966**	0.317		0.683	0.481	**0.800**

Precision, recall, and F1-score. Values > = 0.7 are highlighted

*MC* Molecular classification

**Fig. 2:**
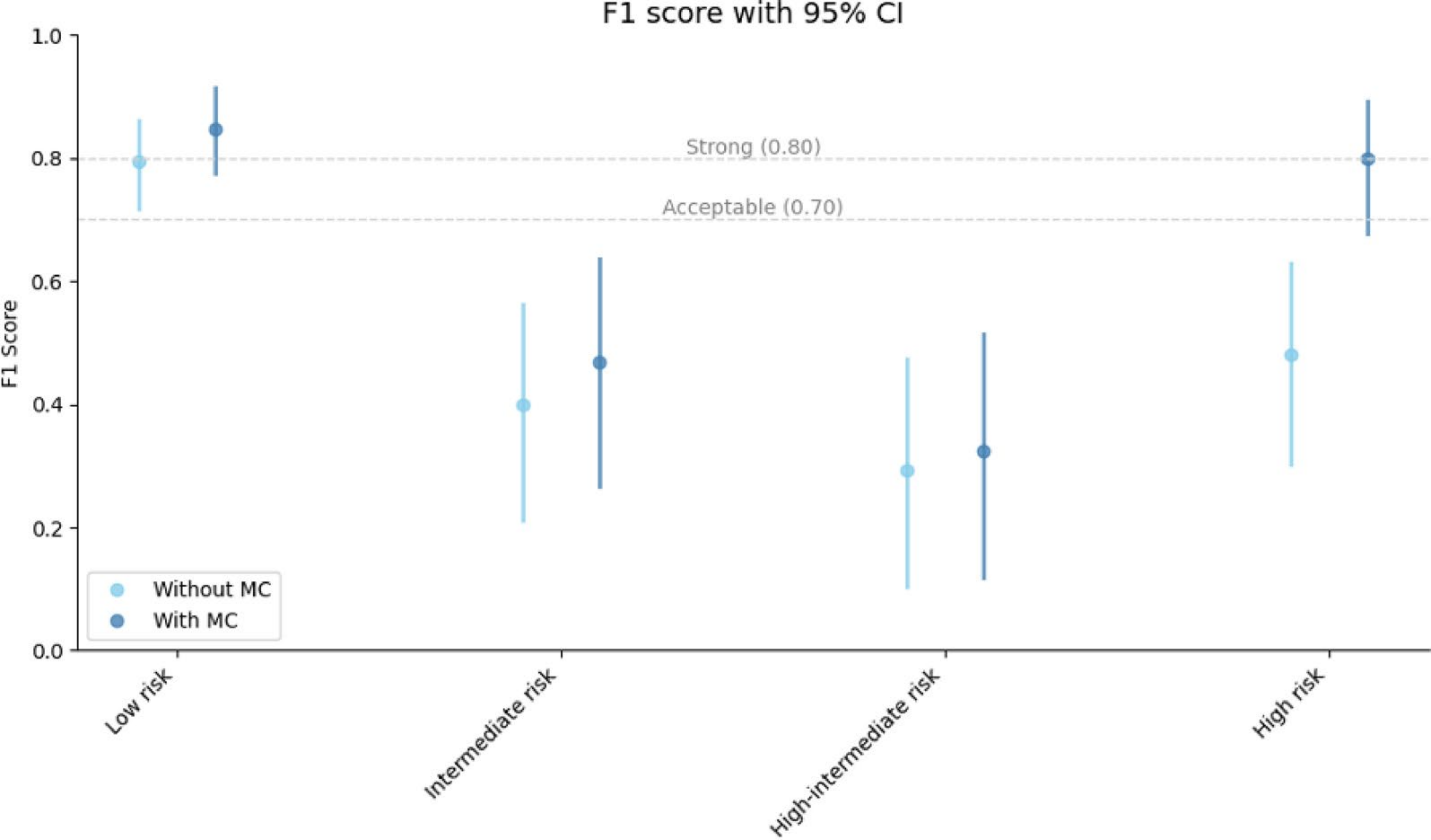
Prediction F1-score with confidence intervals across severity levels

Positive shifts occurred in most measures. A statistically significant shift in F1-score was observed in high risk group, as shown in Fig. 2, including confidence intervals.

The obtained values indicate an improvement in prediction accuracy for the low-risk group, shifting the result from the acceptable range to the strong range. For the intermediate and high-intermediate risk group, only a slight increase was observed; however, both remained below the acceptable threshold. A statistically significant improvement was seen in the high-risk group, where the use of MC improved the results from below the acceptance level to the strong range.

## Discussion

This analysis demonstrated that incorporating MC into preoperative staging significantly enhances the prediction of preoperative risk stratification in endometrial cancer patients, according to ESGO/ESTRO/ESP 2021 guidelines. The most significant improvement was observed in predicting the high-risk group, while MC also enhanced the accuracy in identifying the low-risk group preoperatively. However, distinguishing the intermediate and high-intermediate risk groups preoperatively remains challenging, even with MC. Without MC prior to surgery, there was a tendency to underestimate risk stratification (Table 3).

Molecular features are known to provide better risk stratification than standard histopathology, however, prior studies have focused on postoperative risk groups or novel classifications, making direct comparisons to our results difficult. For example, a small prospective study found that MC changed final risk group in 10.3% of patients [12]. The study was designed to evaluate standard definitive histopathological risk assessment and the additional value of molecular classification.

Another study evaluated the ability of demographic and sonographic variables, along with the ProMisE (Proactive Molecular Risk Classifier for Endometrial cancer) classification, to predict tumor recurrence or progression in 339 women with endometrial cancer. It found that a tumor size of less than 2 cm combined with p53 wild-type status identified approximately 50% of women at very low risk for recurrence or progression [13].

In a study of 658 patients, p53, combined with imaging tests, was found to be a reliable preoperative indicator of advanced disease [14]. However, unlike our study, the investigators included preoperatively advanced stages of EC according to imaging methods, which are already considered high risk regardless of molecular classification. We focused only on the preoperative early stages of EC, where the specific histopathological and molecular features can play a key role in risk stratification.

Our findings can also be viewed in light of the recent commentary by Betella et al., who reported that molecular classification changed FIGO 2023 stage in only 6% of 381 EC patients, meaning about 17 patients needed testing to change treatment for one [15]. In our study, molecular classification led to risk group reclassification in 14% of cases. This higher reclassification rate is likely due to the use of preoperative data, where not all parameters are fully known or accurately assessed. Consequently, adding molecular classification only at final histology typically results in fewer changes. While our findings support the added value of molecular profiling for preoperative risk stratification, the clinical and economic implications of routine use remain important considerations.

Although molecular classification improved preoperative risk stratification prediction in our study, 26.6% of patients were misclassified due to factors like LVSI, cervical and myometrial invasion, and lymph node metastasis, particularly in the intermediate and high-intermediate risk groups. Although this was not the primary focus of our study, it represents a relevant secondary observation that further highlights the limitations of preoperative assessment. Substantial LVSI is a strong prognostic factor that can upstage a case, but it is typically identified only in the definitive surgery specimen. It is important to note that the definition of substantial LVSI is not standardized internationally, with varying criteria used across guidelines [16]. In our study, we use the ESGO/ESTRO/ESP definition of substantial LVSI: diffuse or multifocal involvement of lymphovascular spaces or the presence of tumor cells in five or more lymphovascular spaces [2]. LVSI remains one of the last factors playing a key role in definitive risk stratification, which can not be assessed preoperatively.

Another source of pre- and postoperative bias arises from discrepancies in the assessment of myometrial (sensitivity 81%, specificity 82%) [17] and cervical invasion (sensitivity 63%, specificity 91%) [18] between preoperative imaging (ultrasound) and definitive histopathology. Although ultrasound shows high specificity (98%) for detecting lymph node metastasis, which aids in selecting candidates for debulking surgery rather than SLNB or systemic lymphadenectomy, its low sensitivity (41%) results in frequent postoperative upstaging [19].

Molecular analysis on preoperative biopsy samples is not only feasible but also shows excellent concordance with final pathology [20]. Incorporating this analysis into preoperative assessment can inform individualized surgical strategies. For instance, in cases where sentinel lymph node biopsy fails perioperatively, molecular classification may guide the decision to proceed with side-specific lymphadenectomy. However, the integration of MC into surgical planning introduces new challenges that warrant careful consideration and consensus among international endometrial cancer experts.

For example, preoperative knowledge of *POLE* mutation status could reclassify high-risk tumors (e.g., deep myometrial or cervical invasion, high grade) into the low-risk group, possibly avoiding lymphadenectomy in SLNB failure. However, lymph node status remains crucial for the *POLE*-mutated group, as there’s no evidence on the prognosis of stage III-IV tumors without adjuvant treatment [21]. On the other hand, a large multicenter study showed that preoperatively staged I-II *POLE*-mutated tumors had the lowest frequency of lymph node metastasis compared to other risk groups [22].

Secondly, a *TP53* mutation in tumors with myometrial invasion automatically classifies patients as high-risk, requiring the same adjuvant treatment regardless of lymph node status. Since lymphadenectomy is primarily for staging purposes (rather than a therapeutic one [23]), its necessity in this group is questionable. Furthermore, lymph node metastasis in p53abn tumors has been shown to carry the same prognosis (risk of recurrence and disease-specific death) as in lymph node-negative cases [24]. This suggests that lymph node status has no prognostic value in endometrial carcinomas with TP53 mutation.

Current guidelines suggest that lymph node staging can be waived for tumors without myometrial invasion [2]. However, for *TP53*-mutated tumors limited to the endometrial polyp, without myometrial invasion, adjuvant treatment is not recommended due to the excellent prognosis. Nevertheless, complete staging (including lymph nodes) must be performed as a significant risk of occult lymph node involvement may upstage these tumors to stage III [21]. Therefore, knowing p53 status is crucial for preoperatively low-risk tumors confined to the endometrium. If the tumor is *TP53*-mutated, SLNB should be performed, and in the case of failure, side-specific lymphadenectomy should be considered.

To our knowledge, this is the first study to address the additional value of molecular classification in preoperative stratification into ESGO/ESTRO/ESP risk groups. A statistically significant effect of adding molecular classification to preoperative stratification was observed based on the available data. However, the retrospective design may introduce biases, including information bias from incomplete or inaccurate data and selection bias due to patient selection based on data availability. Despite including only patients with complete data, the retrospective nature limits the ability to draw definitive conclusions. Additionally, as a single-center study, results may be influenced by institutional and personal factors affecting clinical practice.

Incorporating molecular classification into preoperative patient management enhances risk stratification and offers the potential to individualize surgical strategies. As discussed, preoperative identification of *POLE*mut tumors may support omitting lymphadenectomy even in cases of SLNB failure, while *TP53*mut tumors may justify lymph node staging despite limited invasion due to their high-risk profile. These molecular insights can influence whether lymphadenectomy is pursued, the interpretation of nodal involvement, and the overall extent of surgical staging. At the same time, expert ultrasonography remains a key tool for evaluating tumor invasion and guiding surgical planning, despite its known limitations. Together, imaging and molecular classification form a complementary basis for preoperative decision-making. However, their integration introduces new challenges that require further discussion and consensus within the gynecologic oncology community.

## Conclusions

This study found that incorporating molecular classification into preoperative staging improved risk stratification prediction according to ESGO/ESTRO/ESP 2021 risk categories in a retrospective cohort. This approach introduces new challenges, particularly in tailoring surgical treatment, which require further discussion. A multicentric prospective study is needed to confirm these findings.

## Supplementary Information

Below is the link to the electronic supplementary material.

Supplementary Material 1

Supplementary Material 2

## Data Availability

All relevant data are included in the manuscript and supplements. The whole DNA sequencing data are not openly available due to reasons of sensitivity and are available from the corresponding author upon reasonable request. Data are located in controlled-access data storage at the database of the Fingerland Department of Pathology, University Hospital Hradec Kralove.

## Abbreviations

ESGO: European Society of Gynaecological Oncology
ESTRO: European Society for Radiotherapy and Oncology
ESP: European Society of Pathology
MC: Molecular classification
SLNB: Sentinel lymph node biopsy
MRI: Magnetic resonance imaging
LVSI: Lymphovascular space invasion
FIGO: International Federation of Gynecology and Obstetrics
TP53: Tumor protein 53
POLE: Polymeraseε
EC: Endometrial cancer
CT: Computed tomography
IHC: Immunohistochemistry
MMR: Mismatch-repair proteins
NGS: Next-generation sequencing
BMI: Body mass index
IETA: International Endometrial Tumor Analysis
WHO: World Health Organization
NSMP: Non-specific molecular profile
ProMisE: Proactive Molecular Risk Classifier for Endometrial cancer
