# Supplementary material for: Preoperative risk stratification in endometrial cancer using ESGO/ESTRO/ESP 2021 guidelines: accuracy with and without molecular classification
Source: BMC Cancer. 2025 Aug 11;25:1302. doi: 10.1186/s12885-025-14741-5 (PMC12337544; doi:10.1186/s12885-025-14741-5)
Supplement: Supplementary file 1 — Supplementary Material 1 [file 12885_2025_14741_MOESM1_ESM.docx]

|  | Precision |  | Recall |  | F1 |  |
| --- | --- | --- | --- | --- | --- | --- |
| Risk group | without MC (CI) | with MC (CI) | without MC (CI) | with MC (CI) | without MC (CI) | with MC (CI) |
| Low risk | 0.720 (0.616,0.820) | 0.789 (0.692,0.882) | 0.885 (0.797,0.957) | 0.918 (0.843,0.982) | 0.794 (0.716,0.863) | 0.848 (0.777,0.912) |
| Intermediate risk | 0.367 (0.182,0.560) | 0.500 (0.278,0.706) | 0.440 (0.238,0.645) | 0.440 (0.238,0.645) | 0.400 (0.217,0.566) | 0.468 (0.267,0.640) |
| High-intermediate risk | 0.240 (0.077,0.417) | 0.286 (0.095,0.480) | 0.375 (0.125,0.636) | 0.375 (0.125,0.636) | 0.293 (0.100,0.465) | 0.324 (0.111,0.513) |
| High risk | 1.000 (1.000,1.000) | 0.966 (0.889,1.000) | 0.317 (0.180,0.463) | 0.683 (0.531,0.822) | 0.481 (0.305,0.633) | 0.800 (0.678,0.895) |

Supplement 2: Prediction accuracy for risk stratification (low risk, intermediate risk, high-intermediate risk, and high risk). Precision, recall, and F1-score, with confidence intervals.

MC = molecular classification, CI = confidence interval
