# Supplementary material for: Preoperative risk stratification in endometrial cancer using ESGO/ESTRO/ESP 2021 guidelines: accuracy with and without molecular classification
Source: BMC Cancer. 2025 Aug 11;25:1302. doi: 10.1186/s12885-025-14741-5 (PMC12337544; doi:10.1186/s12885-025-14741-5)
Supplement: Supplementary file 2 — Supplementary Material 2 [file 12885_2025_14741_MOESM2_ESM.docx]

**Supplement 1** – Detailed immunohistochemistry staining, next-generation sequencing

The tissue specimens were fixed in formalin and processed routinely for histopathology. All endometrial carcinomas were initially diagnosed and/or finally reviewed and confirmed by an expert gynecologic pathologist (J.L.), following the recommendations of the WHO Classification of Female Genital Tumours (5th edition), International Collaboration on Cancer Reporting, International Society of Gynecological Pathologists and ESGO/ESTRO/ESP Guidelines^1–4^.

All tumors were tested for p53 protein and mismatch repair (MMR) proteins (MLH1, MSH2, MSH6, and PMS2) expression using immunohistochemistry, which was performed on 2 μm thick sections of formalin-fixed and paraffin-embedded tissue using immunostainer BenchMark Ultra (Ventana/Roche, Basel, Switzerland) or Omnis (Dako/Agilent, Santa Clara, CA, USA) and the following antibodies according to the manufacturers´ recommendations: p53 (ready-to-use (RTU), clone Bp53-11, Dako/Agilent), MLH1 (RTU, G168-15, Zytomed), MSH2 (dilution 1:100, G219-1129, Cell Marque), MSH6 (1:250, EP49, Bio SB), and PMS2 (RTU, A16-4, Ventana).

Expression of p53 protein was classified as normal/wild-type (heterogeneous expression of variable intensity in variable proportion of tumor cell nuclei) or mutation-type (strong nuclear overexpression in more than 75–80% of tumor cells, complete absence of staining, or cytoplasmic staining). Cases with equivocal results were further tested for *TP53* gene mutations using NGS. Finally, the tumor was classified as p53-abnormal (p53abn) if there was a mutation-type expression of p53 protein or if NGS showed a pathogenic mutation of *TP53* gene^5^.

Regarding MMR status, the tumor was classified as proficient (pMMR) if all four tested markers showed nuclear expression in tumor cells or deficient (dMMR) if there was loss of expression of ≥1 marker. Cases with equivocal results or heterogeneous expression were further tested for microsatellite instability (MSI) using next-generation sequencing (NGS). Finally, the tumor was classified as dMMR if MMR protein expression was lost or MSI testing using NGS yielded positive results^5^.

The DNA was extracted from paraffin-embedded tissue blocks by the Cobas DNA Sample Preparation Kit (Roche Diagnostics GmbH, Mannheim, Germany) according to manufacturer´s protocol. Mutation analysis was performed by multiparalel sequencing (NGS). Indexed Illumina NGS library was constructed from 100 ng tumor DNA by KAPA Evo Plus Kit (Roche Sequencing Solutions, Inc., USA). Hybrid selection was performed with a custom KAPA KAPA HyperChoice MAX 3Mb T1 Library (Roche). The library was designed using genome build hg38 NCBI Build GRCh38.p14, input genomic regions are listed as follows: *AKT1, ALK, APC, ARAF, ARID1A, ARID1B, ATM, ATR, BARD1, BCL2, BRAF, BRCA1, BRCA2, BRIP1, CCND1, CDK4, CDK12, CDKN2A, CTNNB1, CXCR4, DICER1, DDR2, EGFR, EPCAM, ERBB2, ERG, ESR1, ETV1, FANCA, FANCL, FAT1, FGFR1, FGFR2, FGFR3, FH, FOXL2, GNAS1, GNA11, GNAQ, HDAC2, HRAS, H3-3A, H3-3B, CHEK1, CHEK2, IDH1, IDH2, KEAP1, KIT, KRAS, MAP2K1, MDM2, MET, MLH1, MSH2, MSH6, MTOR, MUTYH, MYD88, MYC, NF1, NF2, NOTCH1, NOTCH2, NRAS, NRG1, NTRK1, NTRK2, NTRK3, NUTM1, MYC, PALB2, PDGFRA, PIK3CA, PIK3R1, POLE, PMS2, PTEN, RAD51B, RAD51C, RAD51D, RAD54L, RB1, RET, ROS1, SMARCA4, SDHA, SDHB, SDHC, SDHD, SMAD4, STAT6, STK11, TERT promotor, TP53*, VHL. Microsatelite regions are as follows: msi10 (chr1:31,915,883- 31,916,003), BAT40, msi9 (chr 1: 230,958,307-230,958,427), MONO-27, BAT 25, BAT26, D2S123, NR24, msi13 (chr2: 119,956,827-119,956,947), msi14 (chr2:200,913,887-200,914,007), D3S1029, msi16 (chr3: 112,155,057-112,155,177), msi6 (chr3:140,959,435-140,959,555), msi17 (chr4:38,132,806-38,132,926), chr5:1,295,063-1,295,183, msi1 (chr5:14,484,946-14,485,066), msi18 (chr5:53,062,878-53,062,998), D5S107, D5S346, msi19 (chr6:111,008,022-111,008,142), msi7 (chr6:152,419,546-152,419,666), D7S519, msi20 (chr7:74,753,041-74,753,161), msi8 (chr7:93,271,202-93,271,322), D8S87, msi4 (chr8:102,275,620-102,275,740), msi21 (chr8:129,862,262-129,862,382), msi22 (chr9:99,967,362-99,967,482), D10S196, NR22, D13S175, msi2 (chr13: 27,559,713-27,559,833), HSP110-T17, D13S153, msi3 (chr13: 78,642,168-78,642,288), NR21, msi12 (chr14:30,722,356-30,722,476), BAT34C4, D17S250, D17S588, D17S787, D18S35, D18D69, D18S64, msi5 ( chr18:62,275,247-62,275,367), D18S55, D18S61, msi15 (chr20:38,517,490-38,517,610), D20S100.

Paired-end cluster generation and sequencing was performed according to standard protocols from Illumina, using MiniSeq kits. Sequencing data analysis and variant classification was performed by NextGENe software (Softgenetics) with minimum 5% variant allele frequency filtering, MSI score was calculated by Genovesa software (Bioxsys).

Finally, all tumors were classified as POLEmut, dMMR, p53abn, or NSMP. Multiple classifiers were categorized as follows: POLEmut-p53abn as POLEmut, dMMR-p53abn as dMMR, and POLEmut-dMMR-p53abn as POLEmut^5^.

References

1. WHO Classification of Tumours Editorial Board. Female genital tumours [Internet]. Lyon (France): International Agency for Research on Cancer; 2020 [cited 2025-04-06]. (WHO classification of tumours series, 5th ed.; vol. 4). Available from: <https://tumourclassification.iarc.who.int/chapters/34>.

2. Matias-Guiu X, Anderson L, Buza N, Ellenson LH, Fadare O, Ganesan R, Ip PPC, Palacios J, Raspollini MR, Werner HMJ, Lax SF, McCluggage WG (2024).*Endometrial Cancer Histopathology Reporting Guide. 5th edition*. International Collaboration on Cancer Reporting; Sydney, Australia. ISBN: 978-1-922324-54-2

3. Cho KR, Cooper K, Phil D. Croce S, Djordevic B, Herrington S, Howitt B, Hui P, Ip P, Koebel M, Lax S, Quade BJ, Shaw P, Vidal A, Yemelyanova A, Clarke B, Ellenson LH, Longacre TA, Shih IM, McCluggage WG, Malpica A, Oliva E, Parkash V, Matias-Guiu X (2019) International Society of Gynecological Pathologists (ISGyP) Endometrial Cancer Project: Guidelines From the Special Techniques and Ancillary Studies Group. Int J

Gynecol Pathol 38(Suppl 1):S114–S122

4. Concin N, Creutzberg CL, Vergote I, Cibula D, Mirza MR, Marnitz S, Ledermann JA, Bosse T, Chargari C, Fagotti A, Fotopoulou C, González-Martín A, Lax SF, Lorusso D, Marth C, Morice P, Nout RA, O’Donnell DE, Querleu D, Raspollini MR, Sehouli J, Sturdza AE, Taylor A, Westermann AM, Wimberger P, Colombo N, Planchamp F, Matias-Guiu X (2021) ESGO/ESTRO/ESP Guidelines for the management of patients with endometrial carcinoma.

Virchows Archiv 478:153–190

5. Dundr P, Cibula D, Doležel M, Fabián P, Fínek J, Jirásek T, Matěj R, Petruželka L, Rob L, Ryška A, Švajdler M, Weinberger V, Zikán M (2021) Molecular testing in endometrial carcinoma (Joint recommendation of Czech Oncological Society, Oncogynecological Section of the Czech Gynecological and Obstetrical Society, Society of Radiation Oncology, Biology and Physics, and the Society of Czech Pathologists). Cesk Patol 57:181–187
